# Patients’ health outcomes after an implementation intervention targeting the physiotherapists’ clinical behaviour

**DOI:** 10.1186/s40945-021-00116-z

**Published:** 2021-10-09

**Authors:** Johanna Fritz, Lena Almqvist, Anne Söderlund, Lars Wallin, Maria Sandborgh

**Affiliations:** 1 School of Health, Care and Social Welfare, Mälardalen University, Box 883, SE-721 23 Västerås, Sweden; 2 School of Education, Health and Social Studies, Dalarna University, Falun, Sweden

**Keywords:** Physiotherapy, Implementation, Musculoskeletal pain, Sick leave, Primary healthcare, Patient outcomes, Behavioural medicine

## Abstract

**Background:**

A behavioural medicine approach in physiotherapy has shown positive effects on increased and sustained activities and participation, including reduced sick leave for patients with persistent musculoskeletal pain. The aim of this study was to explore the health outcomes of patients with persistent musculoskeletal pain treated by physiotherapists who had received active compared with passive support when implementing a behavioural medicine approach.

**Methods:**

An explorative and comparative pre−/post-test trial was conducted. A total of 155 patients with musculoskeletal pain ≥4 weeks were consecutively recruited by physiotherapists in primary healthcare who had received active or passive support when implementing a behavioural medicine approach. Data concerning health outcomes for patients were collected using questionnaires before and after the physiotherapy treatment and at half-, one- and two-year follow-ups. Descriptive, non-parametric and parametric bi- and multivariate statistics were used.

**Results:**

There were no differences over time between the patients treated by physiotherapists who had received active compared to passive implementation support regarding pain-related disability, pain intensity, self-rated health, self-efficacy in performing daily activities, catastrophic thinking related to pain, and fear of movement. Significant improvements over time were identified in both groups regarding all variables and the effect sizes were large. The percentage of patients on sick leave significantly decreased in the patient group treated by physiotherapists who had received active implementation support.

**Conclusion:**

It is very important to include patient outcomes when evaluating the implementation of multicomponent interventions. It seems that the implementation method did not play a major role for the patients’ outcomes in this study. Most of the patients’ health outcomes improved regardless of whether they were treated by physiotherapists who had received active or passive support when implementing a behavioural medicine approach. This was likely because the active implementation support was not extensive enough to enable the physiotherapists to sustain the behavioural medicine approach.

**Trial registration:**

The study protocol was retrospectively registered in ClinicalTrials.gov. ID NCT03118453, March 20, 2017.

**Supplementary Information:**

The online version contains supplementary material available at 10.1186/s40945-021-00116-z.

## Background

Persistent musculoskeletal pain inhibits individuals’ ability to participate in daily life activities, such as social activities, exercising, household chores, and working outside the home [1]. Back pain is the most common location of musculoskeletal pain and is one of the leading causes of disability globally [2]. Work incapacity is strongly associated with persistent pain [3]. A behavioural medicine approach in physiotherapy has shown positive effects on increased and sustained participation in daily life activities, including reduced sick leave for patients with persistent musculoskeletal pain [4–7].

A majority of individuals with persistent musculoskeletal pain seek or are referred to physiotherapy at the primary health care level [1, 8]. To support the implementation of a behavioural medicine approach in primary health care physiotherapy, a six-month active implementation support (AIS) was conducted with a group of physiotherapists and compared with another group of physiotherapists receiving passive implementation support (PIS). There were no significant differences between the two groups of physiotherapists regarding baseline characteristics, see Table 1. The AIS consisted of facilitation comprising multifaceted methods including outreach visits, peer coaching, educational materials, video feedback, individual goal setting, self-monitoring in a diary, prompting managers’ supportive attention to the physiotherapists, and access to a patient information leaflet about what to expect during the physiotherapy session. The PIS consisted of access to a book describing the application of the behavioural medicine approach in physiotherapy. The implementation support has been further described elsewhere [9]. After the period of implementation support, there was an immediate significant change in the physiotherapists’ clinical behaviour in the AIS group but not in the PIS group; however, these changes were not sustained at 6- or 12-month follow-ups [9]. The behavioural medicine approach has been previously evaluated as a multifaceted approach, and it is not clear whether some components are more crucial than others [10]. It is therefore possible that even a small difference in the physiotherapists’ treatment approach could have generated added improvements in health outcomes for the patients treated by the physiotherapists in the AIS group compared to the patients treated by the physiotherapists in the PIS group, which is the focus of this article.

**Table 1:** Characteristics of the physiotherapists in the active implementation support (AIS) group and the passive implementation support (PIS) group

Characteristics	AIS group ***(n = 15)***	PIS group ***(n = 9)***
Sex, male (M)/female (F)	M = 5, F = 10	M = 3, F = 6
Age (years), median (min-max)	37 (23–63)	39 (24–57)
Years of work in primary health care, median (min-max)	9 (1–30)	3 (0.5–16)
Number of physiotherapists with previous behavioural medicine education	13	7

Although patients are often end-users of the innovative methods implemented in physiotherapy, studies have rarely assessed patient health outcomes when implementing a behavioural medicine approach or other clinical guidelines in physiotherapy [11]. In the few studies detected, no differences regarding disability, physical function, pain intensity, sick leave, coping strategies or satisfaction were identified between patients of physiotherapists in the intervention group compared with those in the control group [12, 13]. Therefore, we found it important to also evaluate patient outcomes in our trial to shed light on whether the innovative methods reach the patients after implementation efforts. Thus, the aim of this study was to explore the health outcomes of patients with persistent musculoskeletal pain treated by physiotherapists who had received active compared to passive support when implementing a behavioural medicine approach.

## Methods

The Transparent Reporting of Evaluations with Non-randomized Designs (TREND) statement [14] and the Standards for Reporting Implementation studies (StaRI) [15] were used to report this study (see Additional files 1 and 2).

### Design

An explorative and comparative trial with follow-ups was conducted comparing health outcomes of patients.

### Participants and settings

**Fig. 1:**
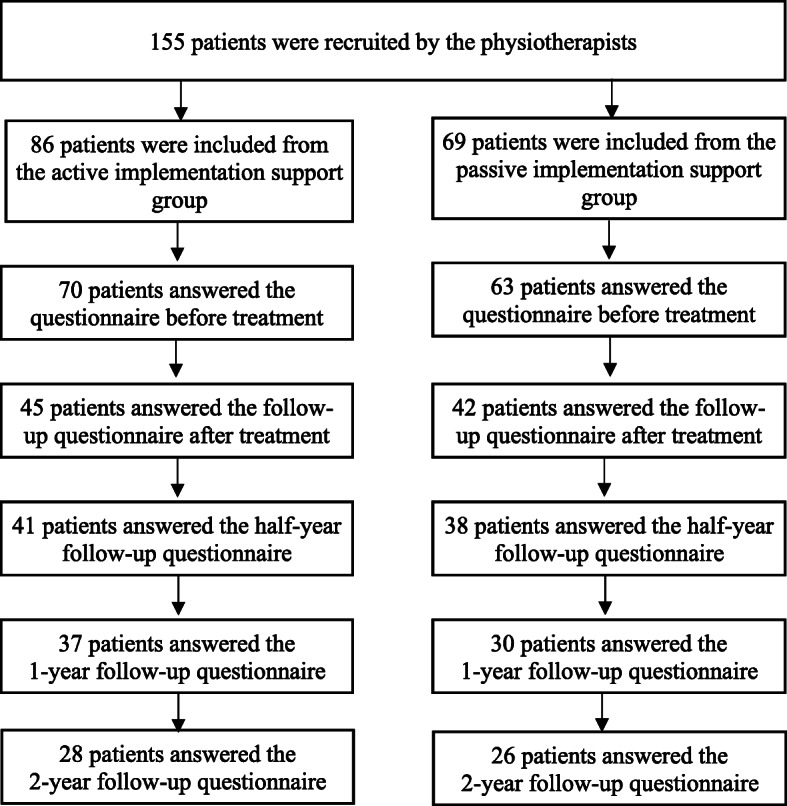
The number of participating patients at each measurement point

The study took place at primary healthcare clinics in three county councils of similar sizes located in the middle of Sweden. In Sweden, primary health care is funded by taxes and patients have direct access to physiotherapy without the need for a referral from a physician. Patients with musculoskeletal pain ≥4 weeks, aged 18–65 years, and who were treated by physiotherapists who received active or passive implementation support were investigated. The exclusion criteria were patients with systemic disease, malignity, serious spinal pathology, osteoarthritis awaiting surgery, diagnosed depression or neurological disease or injury that severely affected activity capacity. The physiotherapists in the AIS group (15 physiotherapists working at seven primary health care clinics) and the PIS group (nine physiotherapists working at seven other primary health care clinics) were asked to consecutively recruit patients during the first one and a half years after the end of the implementation intervention in November 2016 (see Fig. 1). The patients were blinded to their assigned group. A total sample of 230 patients was planned to be included based on a priori power analyses of differences in pain-related disability and expected attrition. To prevent the physiotherapists from including a disproportionate number of patients, a maximum of 15 patients per physiotherapist was established. A total of 155 patients gave their informed consent to participate in the study: 86 patients treated by the physiotherapists in the AIS group and 69 treated by the physiotherapists in the PIS group. The physiotherapists were instructed but failed to report the number of patients who declined participation. Figure 1 illustrates the number of participants who answered the questionnaire at each measurement point.

The characteristics of the included patients are presented in Table 2. There were no significant differences between the patients treated by the physiotherapists in the AIS and PIS groups regarding the characteristics before treatment. The participants lost to follow-up differed from the remaining participants in that they were younger and comprised a larger proportion of men. No differences were found regarding pain duration, pain location and number of treatment sessions.

**Table 2:** Characteristics of the included patients before treatment. AIS = Patients treated by physiotherapists in the active implementation support group. PIS = Patients treated by physiotherapists in the passive implementation support group

Characteristics	AIS group (***n*** = 70)	PIS group (***n*** = 63)
Sex (%), male (M)/female (F)	M = 21.7, F = 78.3	M = 22.2, F = 77.8
Age (years), mean (SD)	47.0 (11.8)	47.4 (11.2)
Number of treatment sessions, mean (SD)	5.1 (5.1)	5.5 (4.4)
Pain duration (%)		
- 1–2 months	16.4	23.3
- 3–6 months	26.2	23.3
- 7 months-2 years	16.4	25.0
- 2–5 years	21.3	15.0
- > 5 years	19.7	13.3
Pain location (%)		
- multiple	63.8	52.5
- lower limb	13.0	23.0
- upper limb	11.6	8.2
- back pain	5.8	9.8
- neck	4.3	3.3
- other	1.4	3.3

### The behavioural medicine approach

The behavioural medicine approach is based on operant conditioning [16], social cognitive theory [17], and cognitive behavioural principles [18]. The approach involves identifying and managing biopsychosocial and lifestyle barriers of importance for the targeted behaviour change [10, 19]. In physiotherapy, for the management of musculoskeletal pain, psychosocial factors refer to patient’s beliefs about the nature of pain, fear, pain catastrophizing, self-efficacy, social interactions, and contextual factors that influence behavioural responses [10, 19]. The biomedical factors refer to the patient’s physical conditions and anatomical dysfunctions. The clinical information is synthesized in an individual functional behavioural analysis about potential causal relationships between biopsychosocial factors and the behavioural performance [10, 19]. Behaviour change techniques are important tools and include, for instance, the patient’s goal setting, self-monitoring of behaviour, the setting of graded tasks, problem solving, feedback on the patient’s behaviours, and maintenance strategies [10, 19, 20]. Thus, the assessments and treatments are individually tailored in terms of content and quantity.

### Data collection

Recommended outcome measures for persistent pain clinical trials were used [21]. The outcome measures involved health, specifically, pain-related disability in performing activities in daily life, pain intensity, self-rated health, sick leave, self-efficacy in performing daily activities, catastrophic thinking related to pain, and fear of movement. In addition, the patients were asked about demographic data and screened for depression. The 2-item Patient Health Questionnaire [22] was used to screen for depression. The two items concern the frequency of symptoms of a depressed mood, scored as 0 (not at all) to 3 (nearly every day). A threshold score of two or more on at least one of the two items concerning depression indicates depression [22]. The patients self-reported their perception of the treatment content based on default options (see Fig. 3), targets for goal setting and goal attainment rated on an 11-graded numerical rating scale, where 0 = no attainment and 10 = total attainment. To interpret the results of a treatment study, the treatment integrity, i.e., how treatments are delivered, is of importance [23]. One way of doing so is to ask patients about the perceived treatment content. Data were collected at the beginning and end of the patients’ treatment period and at the half-, one-, and two-year follow-ups. The treatment content was only asked for after the end of the treatment period. The patients received the first questionnaire from the physiotherapist, including a stamped and addressed envelope in which to return the questionnaire. Reminder e-mail and text messages were sent twice if the questionnaire was not returned. The follow-up questionnaires were mailed to the patients by the researcher, and the same reminder process was used. To encourage the patients to return the first follow-up questionnaire, a lottery ticket was sent as a reward when the questionnaire was returned.

Pain-related disability in performing activities in daily life was measured with the Pain Disability Index (PDI) [24]. The PDI consists of seven questions concerning family/home responsibilities, recreation, social activity, occupation, sexual behaviour, self-care, and life-supporting activities. An 11-graded numerical rating scale is used where 0 = no disability and 10 = total disability. The total index ranges between 0 and 70; the higher the index is, the greater the respondent’s disability due to pain. The PDI has been reported to be valid and reliable [24–26], and its internal consistency reliability for the current study was good (α = .89).

The estimated average pain intensity during the previous week was measured with an 11-graded numerical rating scale where 0 = no pain and 10 = worst possible pain [27]. The numerical rating scale is reported to be valid and reliable for measuring pain intensity [28].

Self-rated health was measured with the EuroQoL visual analogue scale (EQ VAS) [29]. Health status is rated on a visual analogue scale from 0 to 100, where 0 = the worst imaginable health and 100 = the best imaginable health. The validity and reliability of the EQ VAS are acceptable [30].

The proportion of patients who were on sick leave was measured with self-reports of shorter sick leave (≤ 14 days) and register data from the Swedish Social Insurance Administration. The self-reported sick leave data were validated in relation to the register data from the Swedish Social Insurance Administration. The register data were used if the sick leave data were inconsistent or missing.

Self-efficacy in performing daily activities was measured with the Functional Self-efficacy Scale (SES) [31]. The SES consists of 20 questions concerning confidence in the ability to perform activities in everyday life, such as carrying out the trash or driving a car, despite pain. An 11-graded numerical rating scale is used where 0 = not at all confident and 10 = very confident. The total index ranges between 0 and 200, and the higher the index is, the higher the respondent’s self-efficacy due to pain. The SES is viewed as a valid and reliable instrument [32, 33], and its internal consistency reliability for the current study was very good (α = .96).

Catastrophic thinking related to pain was measured with the catastrophizing subscale of the Coping Strategies Questionnaire (CSQ) [34]. The subscale comprises six statements about what people think or do when they have pain. The respondent’s agreement with each statement is rated on a scale from 0 to 6, where 0 = never think or do that and 6 = always think and do that. The total index ranges between 0 and 36, and the higher the index, the higher the respondent’s catastrophic thinking related to pain. The catastrophizing subscale is reported to be valid and reliable [35].

Fear of movement was measured with the 11-item Tampa Scale of Kinesiophobia (TSK-11) [36], which comprises 11 statements about people’s experiences of pain. The respondent’s agreement with each statement is rated on a scale from 1 to 4, where 1 = strongly disagree and 4 = strongly agree. The total index ranges between 11 and 44, and the higher the index, the higher the respondent’s fear of movement. The TSK-11 is reported as a valid and reliable instrument [36].

### Data analyses

A formal normality test [37] showed that assumptions of normality were acceptable (z-value < 3.29) for data concerning pain-related disability, pain intensity, self-rated health, catastrophic thinking, and fear. All data, except for sick leave, were analysed using descriptive statistics and both parametric and non-parametric statistics. Because the parametric and non-parametric tests corresponded with regard to the findings, only the results of the parametric tests are reported. In those cases when data was missing from at least one measurement point, all data from the patient were excluded and handled as missing data. A two-way mixed analysis of variance (ANOVA) [38, 39] was used to analyse within- and between-group differences. Greenhouse-Geisser and Huynh-Feldt procedures were used to correct for no compound symmetry [39]. To identify when the changes occurred, the least significant difference post hoc test was performed [39]. Pre-values were compared with post- and follow-up values. The effect size was calculated using partial eta-squared η_p_^2^, where η_p_^2^ = .01 represents a small effect, η_p_^2^ = .06 represents a medium effect and η_p_^2^ = .14 represents a large effect [40].

The proportion of patients on sick leave at measurement points is accounted for in percentages. Changes over time were analysed using Cochran’s *Q* test and McNemar’s test for post hoc calculations [38]. Between-group differences were calculated using the Chi-square test and Fisher’s exact test when *n* < 5 [41].

Demographics and treatment content are accounted for in percentages, mean values and standard deviation, and median, maximum, and minimum values. Differences between the groups were calculated using Pearson’s Chi-square test, the independent *t* test, and the Mann-Whitney *U*-test [39]. The significance level was set at *p* ≤ .05 for all tests. All analyses were performed on a per-protocol basis. The IBM Statistical Package for the Social Sciences (SPSS) for Windows, version 26, was used for all analyses.

## Results

There was no significant difference between the patients treated by the physiotherapists in the AIS and PIS groups regarding depression (item 1: *p* = .52, item 2: *p* = .15), indicating that there was no need to control for depression in the analysis. A total of 29.0% of the patients in the AIS group and 39.7% of the patients in the PIS group rated 2 or 3 on at least one of the depression items.

**Fig. 2:**
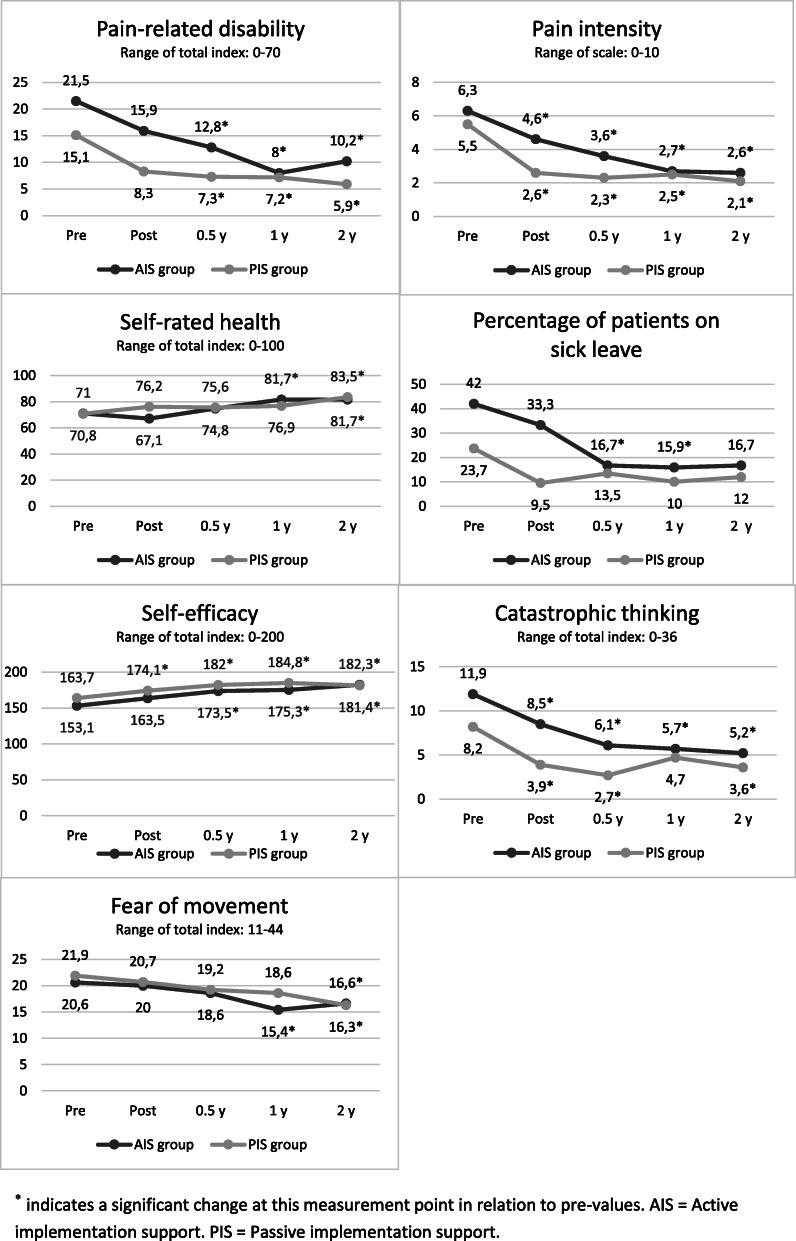
Mean values for the health outcomes for the patients treated by the physiotherapists in the active and passive implementation support groups before and after the treatment period and at half-, 1- and 2-year follow-ups

There were no significant differences between the patients treated by physiotherapists in the AIS and PIS groups regarding pain-related disability in performing activities in everyday life, pain intensity, self-rated health, self-efficacy in performing daily activities, catastrophic thinking related to pain, and fear of movement over time (see Table 3). When exploring the differences over time in each group separately, there were significant improvements in both groups regarding all these variables and the effect sizes were large (see Table 3). Figure 2 illustrates descriptive data and the measurement points at which the changes were identified.

**Table 3:** Interactions of changes in health outcomes over time and between groups, and changes over time and effect sizes for the AIS and PIS groups separately. *AIS* Active implementation support. *PIS* Passive implementation support

	Interaction over time x group		Changes over time *AIS group*			Changes over time *PIS group*		
	***F*** (df_time x group_, df_error_)	***p***	***F*** (df_time_, df_error_)	***p***	η_p_^2^	***F*** (df_time_, df_error_)	***p***	η_p_^2^
**Pain-related disability** (*n* = 42)	.72 (2.7, 108.9)	.53	5.97 (2.4, 55.0)	<.01	.21	3.33 (2.9, 48.8)	.03	.16
**Pain intensity** (*n* = 39)	1.26 (4, 148)	.29	15.08 (4, 92)	<.01	.40	9.42 (4, 56)	<.01	.40
**Self-rated health** (*n* = 41)	1.46 (2.6, 105.6)	.23	3.95 (2.1, 45.6)	.03	.15	4.15 (4, 72)	<.01	.19
**Self-efficacy** (*n* = 39)	.43 (2.7, 100.4)	.71	4.80 (2.6, 58.7)	.01	.17	3.81 (2.5, 34.4)	.03	.21
**Catastrophic thinking** (*n* = 36)	.93 (4, 136)	.45	7.10 (4, 80)	<.01	.26	3.18 (4, 56)	.02	.19
**Fear avoidance of movement** (*n* = 41)	.90 (2.8, 108.0)	.44	7.95 (2.4, 52.2)	<.01	.27	3.50 (2.6, 45.0)	.03	.17

The proportion of patients on sick leave significantly decreased over time in the AIS group *X*^*2*^(4) = 11.88, *p* = .02., but not in the PIS group *X*^*2*^(4) = 3.33, *p* = .50 (see Fig. 2). There were significantly more patients on sick leave in the AIS group than in the PIS group before X^2^(1) = 4.78, *p* = .03 and after *X*^*2*^(1) = 7.35, *p* = .01 the treatment period.

### Treatment integrity

Approximately half of the patients (49% in both the AIS group and the PIS group) reported that treatment goals were set. Among these goals, half (50% in the AIS group and 48% in the PIS group) concerned behaviours such as being able to walk a dog, play golf or do exercises. The other half (50% in the AIS group and 52% in the PIS group) concerned body functions, such as decreased pain or increased muscle strength. The median goal attainment was 7.0 (4.5–9.0) in the AIS group and 7.0 (5.0–9.3) in the PIS group, indicating no difference between the groups (*p* = .53).

**Fig. 3:**
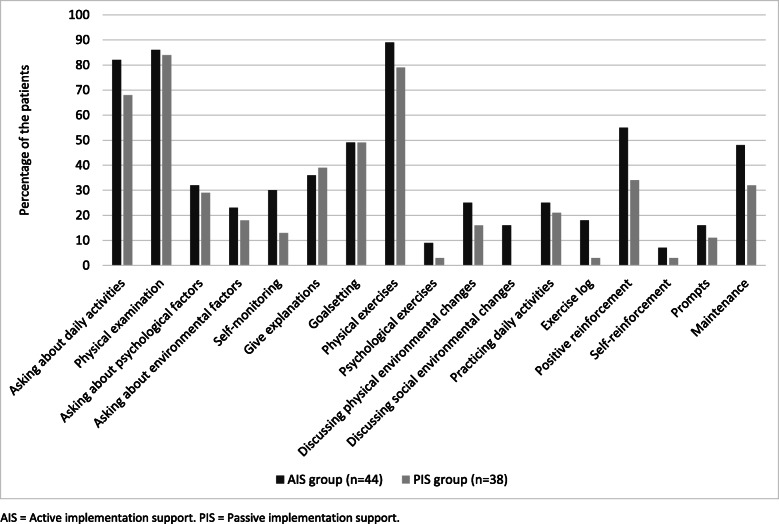
The treatment content reported by the patients treated by the physiotherapists in the active and passive implementation support groups

The most commonly used treatment components in the AIS group, reported by at least half of the patients, were ‘asking about daily activities’, ‘physical examination’, ‘goal setting’, ‘physical exercises’, ‘using positive reinforcement’, and ‘discussing maintenance’. The corresponding treatment components in the PIS group were ‘asking about daily activities’, ‘physical examination’, ‘goal setting’, and ‘physical exercises’ (see Fig. 3).

## Discussion

Even if no significant clinical behaviour change was observed in the physiotherapists at previously reported follow-ups [9], it was not discussed how this would affect patients’ health outcomes. Although the behavioural medicine approach is evaluated as a multifaceted approach [10], current studies provide limited guidance on the specific components that are the most potent and whether there are additional effects when combining two or several behaviour change techniques [4–7, 42–45]. Thus, knowledge is lacking regarding the need to implement either all components or a part of the behavioural medicine approach to achieve an impact on the patients’ health. It is possible that the physiotherapists in the AIS group used a component or combination of components within the behavioural medicine approach that was significant to support the patients’ return to work. In the same way, it could have been possible that if a significant change had been identified in the physiotherapists, the implemented components would not necessarily have been sufficient to change the patient’s health outcomes. This emphasizes the importance of exploring whether multicomponent interventions affect end-users in a ‘real-world’ clinical setting when both significant and non-significant professional behaviour changes are following implementation efforts.

The results identified a difference regarding sick leave between the patients treated by the physiotherapists in the AIS or PIS group over time. In this study, we were not able to disentangle whether the difference in sick leave was due to more active implementation support. There was a considerably larger proportion of patients on sick leave in the AIS group from the beginning, implying that this group had greater potential for improvement, which likely affected the result. In the AIS group, ‘using positive reinforcement’ and ‘discussing maintenance’ seemed to be used more frequently than in the PIS group, which could indicate some impact of the active implementation support. Further studies with a larger sample are however needed to explore the role of the implementation support for the patients’ outcome regarding sick leave. Despite the active support when implementing the behavioural medicine approach, there were no other differences between the patients treated by physiotherapists who had received active vs. passive implementation support. The following discussion will therefore focus on potential reasons for this.

Previously reported results from evaluations of the implementation of the behavioural medicine approach might contribute explanations regarding the absence of group differences in terms of patient outcomes. The physiotherapists in the AIS group did significantly change their clinical behaviour immediately after the period of active implementation support, but the changes were not sustained at the three-month follow-up [9]. The patients were included up to one and a half years after the implementation intervention was completed. Thus, when most patients were included in the current study, the clinical performance did not significantly differ between physiotherapists in the AIS and PIS groups. This implies that the absence of differences in outcomes between the two patient groups might be explained by the active implementation support not being extensive enough to make the physiotherapists in the AIS group sustain the behavioural medicine approach. Therefore, this conjectured lack of a sustained approach in the AIS group could give the appearance that a similar treatment approach had been provided in both groups. When comparing results in implementation research, it is therefore important to be aware of whether the findings convey immediate or sustained results.

Systematic reviews report that active learning activities, such as feedback on performance, peer review, modelling and interaction with others, based on constructivism and experiential learning theories are more effective at enhancing physiotherapists’ clinical practice than passive learning activities [46, 47]. The active implementation support in our study included the same learning activities and theoretical perspectives. Despite these similarities, there were no differences in outcomes between patients treated by physiotherapists in the AIS vs. the PIS group. A previously performed process evaluation of the active implementation support indicated that different mechanisms govern the initiation and maintenance of clinical behaviour change [48]. With that information on hand, it is relevant to reflect upon the implementation support that was provided in our study. It is likely that the active learning activities reported in the systematic reviews as well as the active implementation support offered in our study mainly supported the initiation of clinical behaviour change. Implementation support also needs to include support for sustaining clinical behaviour change. Potential impact mechanisms for sustaining clinical behaviour change are described [48], but need to be tested in future studies.

According to the treatment content reported by the patients (Fig. 3), it is notable that the physiotherapists in both groups focused on biopsychosocial factors, daily activities, and behaviour change techniques to an almost equal extent. The findings are similar to other studies reporting that physiotherapists have a positive attitude towards a biopsychosocial approach [49–51] and that behaviour change techniques such as goal setting are used regularly [50]. Goal setting was, according to the patients, one of the most commonly used technique in our study as well. The techniques used were well within the physiotherapists’ normal skills and practice. The results indicate that parts of the behavioural medicine approach were already implemented in the physiotherapists’ clinical practice. It is possible that the passive support also contributed to the implementation of the behavioural medicine approach. Thus, the treatment in both groups might have involved comparable elements of a behavioural medicine approach to physiotherapy that could explain the similar patient outcomes regardless of group affiliation.

The behavioural medicine approach is based on operant conditioning and social cognitive theory. Although it is recommended to apply theory when designing and evaluating complex behaviour change interventions [52], theory informed interventions have shown mixed effects [53, 54]. Moderate evidence was reported for operant conditioning-based interventions in reducing long term disability in chronic pain, but conflicting evidence was reported in a subacute population [53]. Social cognitive theory-based interventions were reported no more effective for behaviour change related to physical activity than non-theory-based interventions [54]. Inconsistency in intervention dosage, delivery and trial settings are possible explanations for the varied effects of theory-based interventions. It is therefore not possible to conclude that the theory-base of the behavioural medicine approach contributed to the patients’ improved health outcomes in this study.

When reviewing the baseline findings, the patients included in this study appeared to have moderate psychosocial barriers of importance for pain-related behaviour change. According to Sullivan et al., patients in the risk range for pain catastrophizing, fear of movement and perceived disability have the best effect of psychosocial interventions in physiotherapy [55]. It is possible that the effects of a behavioural medicine approach to physiotherapy treatment were not as pronounced for the patients included in our study as they were for patients with more severe psychosocial barriers for behaviour change. This may also be an explanation for why there were no differences between the outcomes of the patient groups. Therefore, future studies need to ensure that an appropriate target group is included to optimize the effect of the treatment. In our study, the inclusion criteria should have been revised to include psychosocial barriers for behaviour change.

Despite the provided active support when implementing the behavioural medicine approach, the patients did not perceive that the physiotherapists in the AIS group considered psychosocial factors to the same extent as physical factors (Fig. 3). Some behaviour change techniques were barely used at all, such as self-monitoring, self-reinforcement, and prompts; this may be because these techniques were outside of the comfort zone for the physiotherapists. The perceived limited use of these techniques is likely one of the reasons why limited differences were found regarding patients’ health outcomes in the AIS vs. the PIS group. Driver et al. [50] found that physiotherapists experience a need for training in delivering psychosocial interventions. Although the active implementation support included training of these components of the behavioural medicine approach, this does not seem to have been sufficient to achieve sustainable changes in the physiotherapists’ clinical practice [9]. Implementation support that focuses more strongly on the maintenance of behaviour change in physiotherapists might improve the requisites for better patient outcomes.

### Limitation and strengths

The main limitation of this study is the lack of power due to the small sample size. A priori power analyses indicated that a sample of 230 patients was needed. However, seven of the 24 included physiotherapists were not able to recruit any patients because of physiotherapists’ sick leave (*n* = 1), maternity leave (n = 1), changed workplace (*n* = 2), and a lack of interest in continuing participation in the study (*n* = 3). This resulted in the inclusion of 155 patients. Furthermore, we do not know how many patients could have been recruited to the study because of the physiotherapists’ failure in reporting the patients who declined participation. The fact that missing data from at least one of the five measurement points were handled by removing all of the data from that patient in the analyses instead of using imputation of missing data further reduced the sample on which the analyses were based. However, imputation of data in this study would have meant an even larger bias due to the high dropout rate (35%) between the first and second measurements [56]. Given the small sample size, the outcomes should be interpreted and generalized with caution.

In the current study, treatment integrity was assessed by allowing the patients to self-report their perceived content of the treatment sessions. This can be an excessively difficult task for patients. It is possible that the patients did not recognize all efforts made by the physiotherapists, meaning that some treatment content may not have been identified. More patients in the AIS group than in the PIS group perceived that self-monitoring was a part of the treatment content, which correlates with the evaluation of the physiotherapists’ observed clinical behaviour change [9]. However, all observed clinical behaviour changes [9] did not correspond to the treatment content reported by the patients in this study. Further research is necessary regarding the validation of treatment integrity measures [57]. Patients’ self-reports were chosen as a pragmatic method to address treatment integrity without vulnerability to reactivity effects. In addition, patients’ adherence to treatment may have influenced the evaluated outcomes and should preferably be measured in future studies.

The choice of methods for analysing the outcomes were based on assumptions of a normal distribution, sample size, and response scales used in the measures [41]. Visual inspection and formal normality tests using skewness and kurtosis [37] showed that assumptions of normality were acceptable for most, but not all, of the data, and both ratio and ordinal scales were used. Data concerning pain-related disability, self-efficacy, and fear of movement were treated as continuous data because the total index was based on at least seven subcategories [58]. Overall, it was not obvious whether parametric tests other than those for pain-related disability and fear of movement should have been used. Therefore, both non-parametric and parametric tests were used to avoid type 1 and 2 errors.

A strength of our study is the awareness of the risk of type 1 and 2 errors. This study had an explorative design, meaning that any tendencies of changes in patients’ health were of interest. Therefore, a less conservative post hoc test was chosen. This may have increased the risk of type 1 error. Multiple comparisons also increase the risk of type 1 error. To minimize multiple comparisons, a repeated-measures ANOVA is preferred [59]. To control for type 1 error because of violated compound symmetry, corrections with Greenhouse-Geisser and Huynh-Feldt were performed [39]. Although corrections with Greenhouse-Geisser tend to be too conservative with risk for type 2 error [59], this was not the case in the current study because the findings corresponded whether we used the correction or not.

## Conclusions

It is very important to include patient outcomes when evaluating the implementation of multicomponent interventions. The implementation support provided did not appear to play a major role for the patient outcomes in this study. Most of the patients improved their health regardless of whether they were treated by physiotherapists who had received active or passive support when implementing a behavioural medicine approach. The fact that our previous studies have evaluated the implementation process by focusing on the physiotherapists’ clinical behaviour change has contributed to explanations of the patient outcomes in this study. The absence of differences between groups was likely because the active implementation support was not extensive enough to enable the physiotherapists in the AIS group to sustain the behavioural medicine approach. This emphasizes the value of including both professional and patient outcomes in the process evaluation of an implementation intervention.

## Supplementary Information

**Additional file 1.** TREND Statement Checklist.

**Additional file 2.** Standards for Reporting Implementation Studies: the StaRI checklist for completion.

## Data Availability

The datasets used and analysed during the current study are available from the corresponding author on reasonable request.

## Abbreviations

AIS: Active implementation support
PIS: Passive implementation support
PDI: Pain disability index
EQ VAS: EuroQoL visual analogue scale
SES: Functional self-efficacy scale.
ANOVA: Analysis of variance
